# The Risk of Adjacent Vertebral Fracture Following Balloon Kyphoplasty in Patients With Previous Adjacent Vertebral Fracture

**DOI:** 10.7759/cureus.72627

**Published:** 2024-10-29

**Authors:** Koji Matsumoto, Masahiro Hoshino, Hirokatsu Sawada, Sosuke Saito, Tomohiro Furuya, Yuya Miyanaga, Kazuyoshi Nakanishi

**Affiliations:** 1 Department of Orthopaedic Surgery, Nihon University Itabashi Hospital, Tokyo, JPN; 2 Department of Orthopaedic Surgery, Sonoda Medical Institute Tokyo Spine Center, Tokyo, JPN

**Keywords:** adjacent vertebral body fracture, balloon kyphoplasty, hounsfield unit value, osteoporotic vertebral body fracture, previous adjacent vertebral fracture

## Abstract

Purpose

To examine the characteristics of adjacent vertebral fractures (AVF) following balloon kyphoplasty (BKP) in patients with previous adjacent vertebral fractures and assess whether these previous fractures exert a suppressive effect on AVF development subsequent to BKP.

Methods

One hundred and thirty-three patients who underwent BKP were enrolled. 32 patients had experienced previous adjacent vertebral fractures (Group P), while 101 patients served as the control group with no previous adjacent vertebral fractures (Group C). Clinical parameters were compared between Group P and Group C. AVF incidence was investigated, and Hounsfield unit (HU) values in the adjacent vertebral bodies were assessed. Additionally, the P group was divided into two subgroups based on the presence or absence of AVF. Clinical parameters and the difference in the HU values of the adjacent vertebral bodies above and below were compared between the two subgroups.

Results

In the comparison of Group P and Group C, there was no significant difference in AVF incidence (10/29 (34.5%) vs. 24/101 (23.8%), P=0.337). Conversely, the young adult mean (YAM) was found to be significantly lower (62.1±11.1% vs. 72.0±7.5%, P<0.001*), and the number of previous fractures significantly higher (2.2±1.3 vs. 0.5±0.7, P<0.001*) in Group P. In Group P, AVF occurred in 10 of 29 patients, including one case of re-fracture of a previously fractured adjacent vertebral body and nine cases of fracture in a contralateral adjacent vertebral body without previous fracture. In the comparison of previously fractured adjacent vertebral bodies and contralateral adjacent vertebral bodies in Group P, the AVF incidence was significantly lower (1/29 (3.4%) vs. 9/29 (31.0%), p=0.012*), and the HU value was significantly higher (158.6±62.6 vs. 69.6±23.0, P<0.001*) in previously fractured adjacent vertebral bodies. In the comparison between AVF (+) and AVF (-) in Group P, a significant difference was observed in the HU value difference of the adjacent vertebral bodies above and below (AVF (+): 118.7±67.0 vs. AVF (-): 72.7±53.6, P=0.046*).

Conclusion

Previous adjacent vertebral fractures do not suppress AVF occurrence. AVF is less likely to occur in vertebral bodies with previous fractures due to bone sclerosis, but it is more common in the contralateral adjacent vertebral bodies due to bone fragility. In cases with previous fractures in the adjacent vertebral bodies, patients exhibiting a large difference in the HU values of the adjacent vertebral bodies above and below are more likely to develop AVF.

## Introduction

Osteoporotic vertebral fractures represent a significant ailment prevalent among the elderly population, resulting in pain, diminished quality of life (QOL), and heightened mortality rates [1,2]. The incidence of this condition has been reported as 11% among individuals aged 70-79 and 18% among those aged 80 and above, with an annual influx of approximately 1.4 million new cases [3,4].

Balloon kyphoplasty (BKP), a common surgical intervention for osteoporotic vertebral fractures (OVF), is recognized for its ability to mitigate vertebral body height loss, address local kyphosis, alleviate pain, and enhance QOL [5,6]. However, adjacent vertebral fractures (AVF) are widely recognized as a significant complication arising from BKP, with reported incidence rates ranging from 9.9% to 30.8% [5,7,8]. It is reported that numerous risk factors contribute to the development of AVF [7,9-12]. The mitigation of AVF represents a crucial issue for surgeons performing BKP procedures.

In recent years, there have been concerted efforts to implement scoring systems to determine the likelihood of AVF following BKP and to identify cases with a high risk of AVF [7,9,13]. Nonetheless, there are instances where risk assessment remains challenging, even when utilizing these scoring methods. It is known that cases involving previous fractures in adjacent vertebral bodies and bone sclerosis exhibit distinct patterns in the development of AVF [7,13]. We strongly postulate that patients with previous fractures in adjacent vertebral bodies are less prone to AVF development due to the presence of bone sclerosis, thereby making them particularly suitable candidates for BKP. To our knowledge, there have been no comprehensive reports to date that have extensively addressed the incidence of AVF in patients with previous adjacent vertebral fractures.

The objective of this study was to examine the characteristics of AVF following BKP in patients with previous adjacent vertebral fractures and assess whether these previous fractures exert a suppressive effect on AVF development subsequent to BKP. The findings from this investigation will enable surgeons to formulate more efficient treatment strategies for osteoporotic vertebral body fractures.

## Materials and methods

Patients in this retrospective study were selected from among the 261 cases of BKP performed at a single center between 2011 and 2021. A total of 133 patients (29 males and 104 females, with a mean age of 77.6±5.9 years) meeting the following criteria were enrolled in the study: individuals aged 60 or older, and patients who could be followed up for a minimum of two months post-surgery. The exclusion criteria were neuropathy, pathologic fractures, a history of spinal surgery, and the presence of osseous bridges connecting fractured vertebral bodies with adjacent vertebral bodies, such as diffuse idiopathic skeletal hyperostosis. There were 32 cases with previous fractures in adjacent vertebral bodies (Group P), consisting of 18 cases of previous upper adjacent vertebral fractures, 11 cases of previous lower adjacent vertebral fractures, and three cases of previous bilateral adjacent vertebral fractures. The remaining 101 patients, with no previous adjacent vertebral body fractures, served as a control group (Group C).

**Figure 1:**
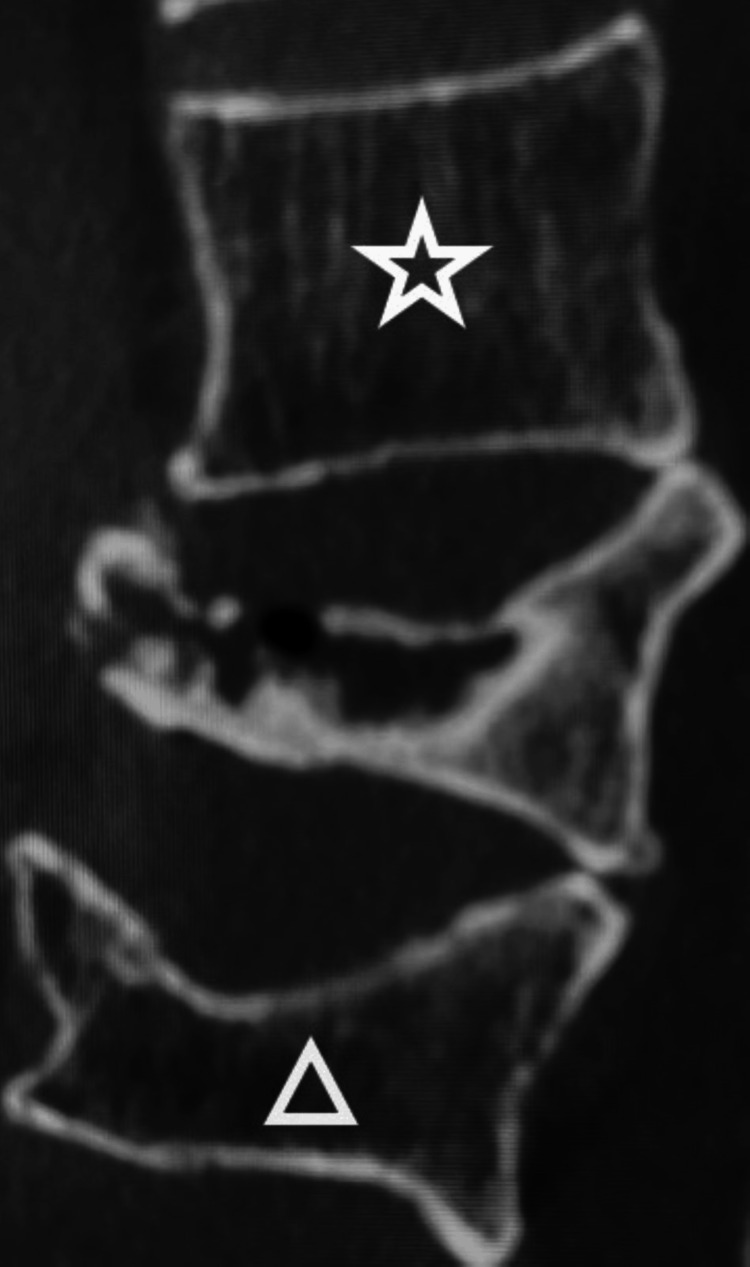
Case of previous fracture in adjacent vertebral body △ indicates previous fracture in adjacent vertebral body. ☆ indicates contralateral adjacent vertebral body without previous fracture.

The incidence of AVF within two months post-surgery and Hounsfield unit (HU) values were compared between previously fractured adjacent vertebral bodies (Figure 1△) and contralateral adjacent vertebral bodies without previous fractures in Group P (Figure 1☆). The correlation between the height of the previously fractured vertebral bodies and the corresponding HU value was explored. The following parameters were also compared between Group P and Group C: age, sex, body mass index (BMI), AVF incidence within 2 months post-surgery [7,13], young adult mean (YAM) values [14], number of previous fractures [7], steroid usage, parathyroid hormone preparations (PTH), osteoporosis medication, local kyphosis [7], and fracture level [9]. Additionally, to evaluate which cases in the P group were more likely to develop AVF, the P group was divided into two subgroups based on the presence or absence of AVF. The above-mentioned clinical parameters and the difference in the HU values of the adjacent vertebral bodies above and below were compared between the two subgroups.

Image assessment

The diagnosis of vertebral body fracture was established based on clinical symptoms involving lower back pain and the presence of high signal intensity on magnetic resonance imaging short tau inversion recovery (MRI STIR) sequences. Local kyphosis was quantified as the angle between the lower endplate of the upper adjacent vertebral body and the upper endplate of the lower adjacent vertebral body [7]. X-ray images were taken before surgery, immediately following surgery, and at intervals of 1, 2, 6, and 12 months thereafter. Additionally, X-rays were taken whenever a patient-reported lower back pain during the study period. Vertebral body height (%) was calculated as follows: (minimum vertebral body height of the previous fractured vertebral body)/(minimum vertebral body height of the adjacent non-fractured vertebral body) x 100.

HU value assessment

**Figure 2:**
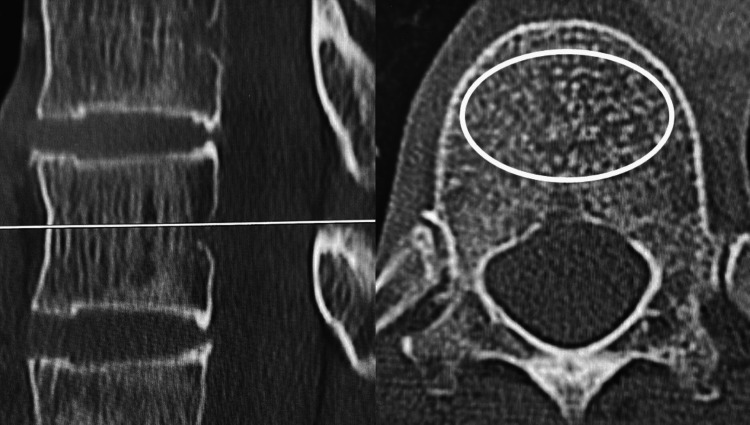
Hounsfield Unit (HU) value assessment An oval-shaped region of interest encompassing as much cancellous bone as feasible was selected from a cross-sectional image of the vertebral body's central portion.

Preoperative computed tomography (CT) imaging was conducted using the Canon Aquilion CX system. HU values were quantified using a picture archiving and communication system (PACS). To obtain measurements, an oval-shaped region of interest encompassing as much cancellous bone as feasible was selected from a cross-sectional image of the vertebral body's central portion. Regions with cortical bone, as well as uneven areas like the posterior venous plexus and bone islands, were excluded from the analysis [15] (Figure 2). To assess intrarater reliability, the HU values were measured three times.

Surgical technique

The procedure was performed with the patient under general anesthesia and in the prone position. A needle was inserted, guided by both frontal and lateral X-ray fluoroscopy views, targeting the inner edge of the pedicle. Subsequently, a guide wire was threaded through, a drill was introduced, and the balloon was carefully positioned. The balloon was then inflated to restore the height of the vertebral body. Afterward, the balloon was removed, and the vertebral body was fully filled with cement. Verification of the cement setting was performed, and the surgical incision was closed. Patients were encouraged to begin mobilizing from the day following surgery and were fitted with a corset.

Statistical analysis

All statistical analyses were conducted using EZR (64-bit). Univariate comparisons between groups were performed utilizing Student's t-test, χ2 test, or Fisher's exact test, as appropriate. Pearson's correlation coefficient was employed to assess the correlation between vertebral height and HU values. A p-value of < 0.05 was considered statistically significant. The receiver operating characteristic (ROC) was utilized to assess AVF following BKP in Group P, and a cut-off value was determined. Additionally, the study measured the accuracy of AVF prediction through the area under the curve (AUC). To assess intrarater reliability, the intraclass correlation coefficient (ICC) was calculated.

## Results

Table 1 shows the fracture level of the vertebral bodies at which BKP was performed. The average follow-up duration was 21.8±16.3 months. AVF occurred in 38 of 133 patients (28.6%) over the entire observation period. Within the first 2 months following surgery, AVF was observed in 34 of 133 patients (25.6%). Notably, 34 of 38 patients (89.5%) had AVF within the first two months following surgery. 

**Table 1:** Fracture level at which BKP was performed

Fracture level	Cases
7th thoracic vertebra (T7)	1
T8	0
T9	5
T10	3
T11	6
T12	41
1st lumbar vertebra (L1)	39
L2	16
L3	13
L4	7
L5	2
Total	133
BKP, balloon kyphoplasty	

In Group P, AVF occurred in 10 of 32 patients (31.3%), including one case of re-fracture of a previously fractured adjacent vertebral body and nine cases of fracture in a contralateral adjacent vertebral body without previous fracture. In the comparison of previously fractured adjacent vertebral bodies and contralateral adjacent vertebral bodies in Group P, the AVF incidence was significantly lower in previously fractured adjacent vertebral bodies (1/35 (2.9%) vs. 9/29 (31.0%), p=0.004*) (Table 2). AVF is less likely to occur in vertebral bodies with previous fractures. Additionally, the HU value was significantly higher in previously fractured adjacent vertebral bodies, indicating bone sclerosis (160.5±57.7 vs. 69.6±23.0, p=0.002*) (Table 2).

**Table 2:** Comparison between previous fractured adjacent vertebral bodies and contralateral adjacent vertebral bodies in Group P *p<0.05. Group P, cases of previous fractures in adjacent vertebral bodies; AVF, adjacent vertebral fracture; HU, Hounsfield unit value.

Variable	Previous fractured adjacent vertebral bodies (n=35)	Contralateral adjacent vertebral bodies (n=29)	p-value
AVF	1 (2.9%)	9 (31.0%)	0.004*
HU value	160.5±57.7	69.6±23.0	0.002*

**Figure 3:**
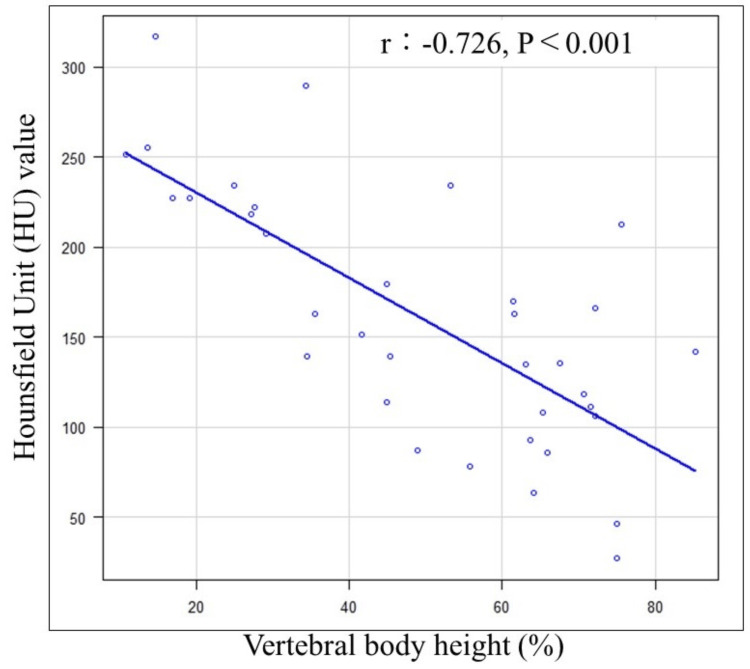
Correlation between vertebral body height and Hounsfield Unit (HU) value A significant negative correlation was observed between vertebral body height and HU value.

In previously fractured adjacent vertebral bodies, a significant negative correlation was observed between vertebral body height and HU value (r=-0.726, p<0.001). As the vertebral body height decreased and compression advanced, the HU value increased (Figure 3). The one case of re-fracture in a previously fractured vertebral body exhibited the lowest HU value and the second highest vertebral body height.

In the comparison of Group P and Group C, age, sex, and BMI exhibited no significant difference (Table 3). However, the YAM value was found to be significantly lower (62.3±10.7% vs. 72.0±7.5%, P<0.001*), and the number of previous fractures significantly higher (2.3±1.4 vs. 0.5±0.7, P<0.001*) in Group P. There was no significant difference in the incidence of AVF (Group P: 10/32 (31.3%) vs. Group C: 24/101 (23.8%), p=0.486) (Table 3), which suggests that previous adjacent vertebral fracture does not suppress AVF occurrence.

**Table 3:** Comparison between Group P and Group C *P<0.05. Group P, cases of previous fractures in adjacent vertebral bodies; Group C, cases of no previous adjacent vertebral body fractures; BMI, body mass index; YAM, young adult mean; AVF, adjacent vertebral fracture

Variable	Group P (n=32)	Group C (n=101)	p-value
Age	79.4±6.5	77.1±5.5	0.134
Sex (Man/Female)	8/24	21/80	0.628
BMI (kg/m2)	22.1±1.9	23.1±3.2	0.196
YAM (%)	62.3±10.7	72.0±7.5	<0.001*
Number of previous fractures	2.3±1.4	0.5±0.7	<0.001*
AVF	10 (31.3%)	24 (23.8%)	0.486
Steroid	2 (6.0%)	4 (4.0%)	0.630
parathyroid hormone preparations	5 (15.2%)	28 (28%)	0.361
Treatment for osteoporosis	11 (30.2%)	45 (44.6%)	0.542
Local kyphosis (°)	0.7±7.6	4.3±8.8	0.120
Fracture level (ThoracolumbarT11-L2/Thoracic, Lumbar)	25/7	77/24	0.813

In the comparison between AVF (+) and AVF (-) in Group P, a significant difference was observed in the HU value of the adjacent vertebral bodies above and below (AVF (+): 118.7±67.0 vs. AVF (-): 72.7±53.6, P=0.046*) (Table 4). From the ROC curve, the AUC was 0.718 (95% confidence interval: 0.496-0.941), with a cut-off value of 158.8, a sensitivity of 50.0%, and a specificity of 95.5%.

**Table 4:** Comparison between AVF (+) and AVF (-) in Group P *P<0.05. AVF, adjacent vertebral fracture; Group P, cases of previous fractures in adjacent vertebral bodies; BMI, body mass index; YAM, young adult mean; HU, Hounsfield unit

Variable	AVF (+) (n=10)	AVF (-) (n=22)	p-value
Age	80.9±5.5	78.7±7.2	0.539
Sex (Man/Female)	3/7	5/17	0.681
BMI (kg/m2)	21.2±2.4	22.5±1.6	0.157
YAM (%)	61.5±13.6	62.6±9.5	0.847
Number of previous fractures	1.8±0.8	2.6±1.7	0.272
Steroid	1 (10.0%)	1 (4.5%)	0.534
parathyroid hormone preparations	3 (30.0%)	3 (13.6%)	0.346
Treatment for osteoporosis	4 (40.0%)	8 (36.4%)	1.000
Local kyphosis (°)	4.8±4.5	-1.2±8.0	0.106
Fracture level (ThoracolumbarT11-L2/Thoracic, Lumbar)	8/2	16/6	1.000
HU value difference of the adjacent vertebral bodies above and below	118.7±67.0	72.7±53.6	0.046*

The intrarater reliability for HU value measurement was assessed, and the ICC was 0.996.

## Discussion

**Figure 4:**
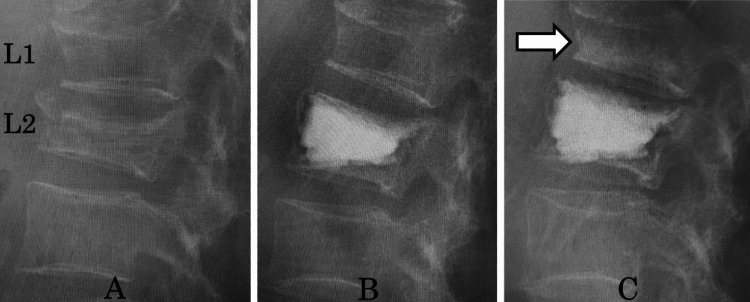
The case which developed adjacent vertebral fracture (AVF) in a previous fractured vertebral body A: Vertebral body fractures on 2nd lumbar vertebra (L2), upper previous adjacent vertebral fracture (L1). B: Balloon kyphoplasty (BKP) was performed on L2. C: AVF in upper previous fractured vertebral body (L1).

AVF was observed infrequently in adjacent vertebral bodies with previous fractures (1/35 (2.9%)). The HU value in previously fractured vertebral bodies was notably higher at 160.5, compared to 69.6 in contralateral adjacent vertebral bodies without previous fractures (Table 2). HU values are associated with T-scores and represent a novel approach to assessing osteoporosis in degenerative vertebrae [15-17]. In addition, HU values are highly effective for assessing local bone strength, offering the significant advantage of selectively measuring specific vertebrae where bone strength is of interest. The threshold range for diagnosing osteoporosis based on vertebral body HU values is reported to be 90.9-138.7 [18]. Notably, the HU value of previously fractured vertebral bodies in our study was considerably higher at 160.5, reflecting bone sclerosis. Considering the observed negative correlation between vertebral body height and HU value in previously fractured vertebral bodies (Figure 3), it is plausible that the HU value increases as the vertebral body collapses, cancellous bone is compressed, and bone sclerosis progresses. Consequently, AVF was less likely to occur in previously fractured vertebral bodies. The one case of AVF in a previously fractured vertebral body exhibited the lowest HU value and the second-highest vertebral body height. In instances where vertebral body compression is mild, fragile cancellous bone persists, leading to the consideration that AVF could still occur even in previously fractured vertebral bodies (Figure 4).

In Group P, the YAM value was significantly lower and the number of previous fractures was significantly higher (Table 3). All of the cases in Group P had at least one previously fractured vertebral body, with an average number of such fractures of 2.3 (Table 3). It has been reported that a previous vertebral fracture increases the risk of a new vertebral fracture by 3-5 times [19,20]. Furthermore, the number of previously fractured vertebral bodies has been identified as a risk factor for AVF after BKP. As the number of previously fractured vertebral bodies increases, the incidence of AVF also rises [7]. Based on these reports, it can be inferred that Group P constitutes a population with pronounced bone fragility and susceptibility to AVF. In fact, the HU value of contralateral adjacent vertebral bodies without previous fractures was 69.6, indicating bone fragility. Therefore, it is likely that AVF occurred in such vertebral bodies.

In the P group, when there was a large difference in the HU values of the adjacent vertebral bodies, AVF was more likely to occur (Table 4). This is believed to be due to a significant difference in bone strength, in which one adjacent vertebral body shows bone sclerosis while the other exhibits bone fragility, leading to stress concentration on the adjacent vertebral body with bone fragility. In such cases, combining fixation may be a treatment option to prevent AVF. Whether fixation surgery is effective in preventing AVF remains a topic for future research.

Three cases with previous fractures in both adjacent vertebral bodies did not develop AVF. Cases with previous fractures in both adjacent vertebral bodies may be less likely to develop AVF and may be good candidates for BKP.

The distinguishing feature of Group P is that previously fractured vertebral bodies are less prone to developing AVF, whereas contralateral adjacent vertebral bodies without previous fractures are more susceptible to AVF. Particularly in cases with a large difference in the HU values of the adjacent vertebral bodies above and below, AVF is more likely to occur.

The limitations of this study include the small sample size, the omission of the evaluation of any clinical outcomes other than AVF, and the fact that this was a retrospective study. In addition, the possibility that other confounding factors may have influenced the results of this study cannot be ruled out.

## Conclusions

Previous adjacent vertebral fractures do not suppress AVF occurrence. AVF is less likely to occur in vertebral bodies with previous fractures due to bone sclerosis, but it is more common in the contralateral adjacent vertebral bodies due to bone fragility. Even in cases with previously fractured vertebrae, AVF can occur when there is minimal vertebral compression. When both adjacent vertebral bodies have previous fractures, AVF is less likely to develop. In cases with previous fractures in adjacent vertebral bodies, AVF is more likely to occur when there is a large difference in the HU values of the adjacent vertebral bodies above and below. Information regarding these characteristics can assist surgeons in formulating appropriate surgical strategies.
